# Ship Fever in Canada

**Published:** 1847-09

**Authors:** 


*Ship Fever in Canada.*—This disease, which is but a malignant modification of typhus, has carried off great numbers; and, we are sorry to add, from its remarkably contagious nature, is rapidly gaining ground in this city, Quebec, and those places along the route to the upper province usually followed by the emigrants. In consequence of the crowded state of the passengers on shipboard, aided by poor diet, want of free ventilation, and that total absence of personal cleanliness, for which the pauper class of the Irish who arrive here appear to be distinguished—numbers have perished on the passage; while in those, whose constitutions have enabled them to weather the hardships and distress incident to protracted voyages under the circumstances mentioned, the disease rapidly develops itself after their arrival, and is characterized by the same malignity, and a nearly uniform mortality, under whatever circumstances treated. When treated early, the disease is most usually found manageable; but if delay takes place, even for a few days, before medical assistance is called in, the cases most frequently turn out unfavorably. It is usually ushered in by a rigor, general *malaise*, and the usual concomitants of a febrile attack; to which are superadded a marked prostration of nervous energy, mental depression, disinclination to motion, the tongue usually affording palpable evidence of irritation or congestion of the mucous membrane of the alimentary canal, and in many cases the icteroid tint of the conjunctiva and skin denoting a congested state of the hepatic system, and an impairment of its functions. Diarrhœa, dysentery, and bronchitis, are the usual complications, and in one respect the disease differs materially from the typhus which in previous years has prevailed among the emigrants, in the supervention of profuse sweats, of a *non-critical* character, breaking out at irregular periods of the disease, and in all the cases in which we have seen it, indicating the necessity for an immediate recourse to a stimulant treatment. Petechiæ make their appearance usually about the sixth day, and are diagnostic of the severity of the disease and its type.

With reference to the treatment, we have to observe that one of an active nature is to be generally avoided. So great is the nervous depression and the debility, that abstraction of blood, either local or general, can seldom be borne with impunity, even in those cases which, under ordinary circumstances, would appear most to demand it. In other respects, the treatment to be pursued is that usually employed in febrile cases, omitting every article likely to induce, or keep up, irritation of the mucous membrane; while a cautious stimulation is usually early demanded when the pulse affords signs of weakness.

Up to the present moment, 32,338 emigrants have landed on our shores; and of this number, exclusive of those who have died on the passage, and are sick in this city, Quebec, and the intermediate places, (and of whom we have no record, because not entering the hospitals,) upwards of 5000 have been known to have been ill—being about one-sixth of the whole number. The medical staff at the Quarantine, and at the Emigrant

Sheds in this vicinity of the city, have received additions to their numbers; but are yet insufficient to meet the exigency. The medical officers in charge are worn out and harrassed with their arduous and unceasing labors. The mortality is appalling. At the Quarantine establishment at Grosse Isle, 2796 cases, of which more than four-fifths were cases of fever, have been treated between the 8th of May and the 19th of June. Of this number 565 have died, being at the rate of 1 in every 4·9 cases, or 20·4 per cent. At the Emigrant Hospitals, in the neighborhood of this city, between the 13th and 28th of June, 1420 cases, all of fever, were admitted—the deaths during the same period numbering 331, affording a greater ratio of mortality, the rate being 1 to every 4·2 cases, or 23·8 per cent.—*British American Journal of Med. and Phys. Science.*

